# The direct and indirect impact of COVID-19 pandemic on maternal and child health services in Africa: a scoping review

**DOI:** 10.1186/s41256-022-00257-z

**Published:** 2022-07-20

**Authors:** Prince A. Adu, Lisa Stallwood, Stephen O. Adebola, Theresa Abah, Arnold Ikedichi Okpani

**Affiliations:** 1 School of Population and Public Health, University of British Columbia, Vancouver, BC Canada; 2 British Columbia Centre for Disease Control, Vancouver, BC Canada; 3 Ladoke Akintola University of Technology (LAUTECH) Teaching Hospital, Ogbomoso, Nigeria; 4 St Paul’s Sinus Centre, St Paul’s Hospital, Burrard St, Vancouver, BC Canada; 5 California State University, Sacramento, CA USA; 6 National Primary Health Care Development Agency, Abuja, Nigeria

**Keywords:** COVID-19, Africa, Maternal and child health, Reproductive health

## Abstract

**Introduction:**

The novel coronavirus disease 2019 (COVID-19) continues to disrupt the availability and utilization of routine and emergency health care services, with differing impacts in jurisdictions across the world. In this scoping review, we set out to synthesize documentation of the direct and indirect effect of the pandemic, and national responses to it, on maternal, newborn and child health (MNCH) in Africa.

**Methods:**

A scoping review was conducted to provide an overview of the most significant impacts identified up to March 15, 2022. We searched MEDLINE, Embase, HealthSTAR, Web of Science, PubMed, and Scopus electronic databases. We included peer reviewed literature that discussed maternal and child health in Africa during the COVID-19 pandemic, published from January 2020 to March 2022, and written in English. Papers that did not focus on the African region or an African country were excluded. A data-charting form was developed by the two reviewers to determine which themes to extract, and narrative descriptions were written about the extracted thematic areas.

**Results:**

Four-hundred and seventy-eight articles were identified through our literature search and 27 were deemed appropriate for analysis. We identified three overarching themes: delayed or decreased care, disruption in service provision and utilization and mitigation strategies or recommendations. Our results show that minor consideration was given to preserving and promoting health service access and utilization for mothers and children, especially in historically underserved areas in Africa.

**Conclusions:**

Reviewed literature illuminates the need for continued prioritization of maternity services, immunization, and reproductive health services. This prioritization was not given the much-needed attention during the COVID-19 pandemic yet is necessary to shield the continent’s most vulnerable population segments from the shocks of current and future global health emergencies.

**Supplementary Information:**

The online version contains supplementary material available at 10.1186/s41256-022-00257-z.

## Introduction

On March 11, 2020, the World Health Organization (WHO) declared a global pandemic as the severe acute respiratory syndrome coronavirus 2 (SARS-CoV-2) spread across the globe [1]. The declaration by the WHO was quickly followed in jurisdictions across the world, by the implementation of national control measures [2, 3]. Some of the most difficult aspects of those measures pertained to health care services [4]. In many instances, health care facilities were closed to ‘routine’ services as part of pandemic mitigation measures [5]. This is a troubling disruption to an already overburdened sector in Africa. African countries had witnessed the devastating impact of communicable disease outbreaks on health service provision and health outcomes [6–8]. Prior to the start of the COVID-19 pandemic, health systems in Africa have been strained from the burden of historic underinvestment [9, 10], which meant that countries were not on track to achieve global human health development goals [11]. Though some progress had been made over the course of the last few decades, the region continued to have some of the poorest maternal, newborn, and child health (MNCH) outcomes [12, 13].

Most notably, the COVID-19 pandemic and subsequent control measures have impacted MNCH care service availability and uptake in Africa. There are reports of widespread avoidance of health care facilities for fear of being diagnosed with COVID-19 or contracting it from health care workers or other patients [14, 15]. Many health facilities are operating at very limited capacity [15, 16], and resources have been diverted away from MNCH to support emergency response activities [17]. The net impact of the pandemic is projected to not only halt progress on improving MNCH outcomes in Africa, but to potentially reverse decades of gains [18]. Added to the foregoing are concerns of the child developmental impact of lockdowns and school closures [17, 19]. It is expected that recovery from the impact of the pandemic on MNCH will be more drawn out in Africa, as vaccine roll-out programs and uptake have lagged relative to high income countries [20, 21].

The impact of the COVID-19 pandemic on the health of women, mothers and children is a subject of several articles and commentaries [15, 22, 23]. To our knowledge, there has not been a scoping review effort, focusing on peer-reviewed literature, to collect documentation of the effect of the COVID-19 pandemic on the availability and demand for MNCH services in Africa. Therefore, this review was conducted to map the current literature on the impacts of the COVID-19 pandemic on maternal and child health in Africa whilst simultaneously identifying any gaps in knowledge, and highlighting the strategies that have been adopted by actors on the continent to sustain maternal and child health service provision and uptake. The following research question was investigated: “What is known from the current literature about the direct and indirect impacts on Maternal and Child Health during the COVID-19 pandemic in Africa?

## Methods

### Protocol and registration

There is no existing protocol for this review. This scoping review was conducted following the Preferred Reporting Items for Systematic Reviews and Meta‐Analyses (PRISMA) extension for Scoping Reviews [24] and the framework proposed by Arksey and O’Malley [25] to map all the evolving literature on our research topic.

### Search strategy

Through a preliminary search and consultation with our team members, the following research question was developed: *How has the COVID-19 pandemic directly and indirectly impacted maternal and child health in Africa?*

A comprehensive search strategy was developed to identify relevant peer-reviewed papers from the following electronic databases: MEDLINE, Embase, HealthSTAR, Web of Science, PubMed, and Scopus. The databases were searched from January 2020 to March 15, 2022. The final search strategy can be found in the Additional file 1. To be included in this review, literature needed to be published in English from January 1, 2020, to March 15, 2022, focus on maternal and child health or a synonym that was used in our search strategy, focus on the impacts from COVID-19, and concentrate on a region in Africa. Studies that were not focused on Africa, but only mentioned an African country or setting were excluded. Reviews, commentaries, opinions and non-empirical studies were also excluded. Quantitative, qualitative, and mixed-method studies were included to capture varying methods and perspectives of the COVID-19 impact on MNCH in Africa.

### Study selection and eligibility criteria

For any identified duplicates, we included the copy with a more complete description. The screening process was executed by two reviewers. The title and abstract of each article were independently screened by the two authors, and each study was marked as ‘yes’, ‘no’ or ‘unclear’. A study was marked as unclear if its relevance to our topic was unclear upon title and abstract screen. All studies marked as ‘yes’ or ‘unclear’ were included for full-text review. All available full-text articles were independently reviewed by the two authors. Any disagreements on the study selection were resolved through discussion between the two review authors and/or with other research team members if needed. Covidence systematic review software was used for deduplication, screening, and data extraction [26].

### Data extraction and items

Twenty-seven peer reviewed articles were included in the review. These articles included review articles, retrospective studies, cross sectional studies and mixed-method studies. A data-charting form was developed by the two reviewers who conducted full text reviews to determine which themes to extract. Preliminary themes were developed after an initial read of the full text articles and themes were discussed amongst all authors.

### Method of synthesis

Two authors coded each article independently and discussed the codes with the team prior to charting them under the appropriate theme. Standard information about each article including title, topic, setting and outcome of interest were recorded in a Microsoft Excel file.

### Data analysis

The main findings and recommendations by the authors of the included studies were tabulated and narrative descriptions of each article were written. All authors reviewed the themes with corresponding narratives to improve readability.

## Results

### Search results

**Fig. 1:**
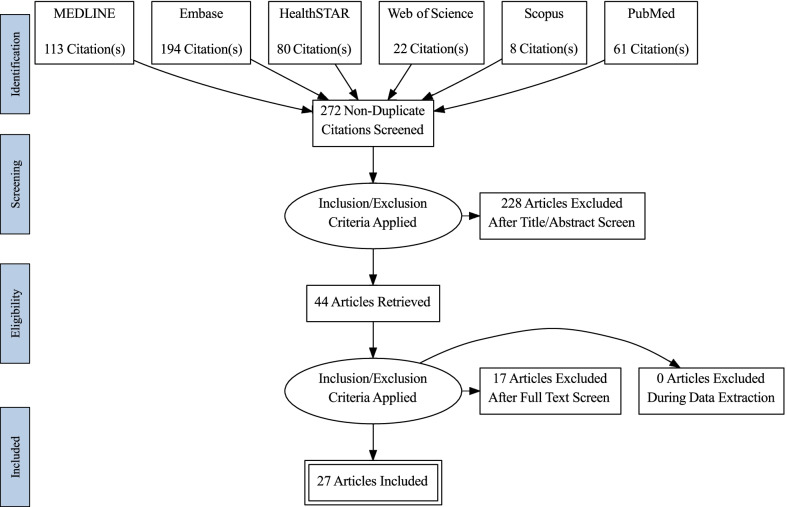
PRISMA flow chart to show the study selection process

Applying the appropriate search filters, a total of 478 articles were identified from our selected databases and 272 articles remained after removing duplicates. After applying the inclusion and exclusion criteria during the title and abstract screening, 44 articles were eligible for full-text screening. After applying the inclusion and exclusion criteria during the full text review, 17 articles were excluded because they were not focused on health service utilization, only mentioned MNCH without an entire focus, did not focus on Africa or only proposed a research protocol on this topic. The characteristics of the remaining 27 articles included in this scoping review are presented in Table 1. The selection process is exemplified in the PRISMA flow diagram (Fig. 1).

**Table 1:** Studies included in the scoping review

Author/year	Title	Journal	Type of study	Setting/country	Outcome of interest	Main message
Abdul-Mumin et al. [27]	Decrease in Admissions and Change in the Diagnostic Landscape in a Newborn Care Unit in Northern Ghana During the COVID-19 Pandemic	Frontiers in pediatrics	Cross-sectional study	Ghana	Neonatal admission and mortality	Substantial decrease in hospital admissions and increases in neonatal mortality during the COVID era
Jensen et al. [28]	Child health services during a COVID-19 outbreak in KwaZulu-Natal Province, South Africa	South African medical journal	Retrospective study	South Africa	Child health services	COVID-19 has led to a disruption in service access and utilization, service delivery and child well-being. There has also been reduction in hospital admissions
Lusambili et al. [29]	"We have a lot of home deliveries" A qualitative study on the impact of COVID-19 on access to and utilization of reproductive, maternal, newborn and child health care among refugee women in urban Eastleigh, Kenya	Journal of migration and health	Qualitative study	Kenya	Access to and utilisation of antenatal care, delivery, and postnatal care	There was reduced utilization of services and delayed care. Preference for home deliveries increased
Balogun et al. [30]	Challenges in access and satisfaction with reproductive, maternal, newborn and child health services in Nigeria during the COVID-19 pandemic: A cross-sectional survey	PLoS ONE	Cross sectional study	Nigeria	Client satisfaction with reproductive, maternal, newborn and child health services	The COVID-19 lockdown posed challenges to accessing RMNCH services for many of the women who were surveyed. Overall satisfaction with care was high
Sayed et al. [31]	Lockdown-associated hunger may be affecting breastfeeding: findings from a large SMS survey in South Africa	International journal of environmental research & public health	Cross-sectional study	South Africa	Breastfeeding, maternal depression and hunger	Breastfeeding initiation rates was high. There was no association between breastfeeding and depressive symptoms
Hailemariam et al. [32]	Exploring COVID-19 Related Factors Influencing Antenatal Care Services Uptake: A Qualitative Study among Women in a Rural Community in Southwest Ethiopia	Journal of primary care & community health	Qualitative study (in-depth interviews and focus group)	Ethiopia	Antenatal care	There was a decline in antenatal care service uptake. Factors associated with the decline include health facility related barriers, perceived poor quality of care during the pandemic, pandemic-related anxiety, and others
Nwafor et al. [33]	Prevalence and predictors of depression, anxiety, and stress symptoms among pregnant women during COVID-19-related lockdown in Abakaliki, Nigeria	Malawi medical journal	Cross-sectional study	Nigeria	COVID-19 related depression, anxiety, and stress symptoms among pregnant women	Depressive symptoms, anxiety and stress were reported by pregnant women during the pandemic. Predictors of depression were multiparity and occupation whiles predictors of anxiety and stress included grand-multiparty, urban residence and trading
Hedstrom et al. [34]	Impact of the early COVID-19 pandemic on outcomes in a rural Ugandan neonatal unit: A retrospective cohort study	PLoS ONE	Retrospective cohort	Uganda	Neonatal admissions and mortality	There was decreased antenatal care. There was an increase newborn mortality during the early days of the COVID-10 pandemic
Asratie et al. [35]	Unintended pregnancy during covid-19 pandemic among women attending antenatal care in northwest Ethiopia: Magnitude and associated factors	International journal of women's health	Cross-sectional study	Ethiopia	Unintended pregnancy	Unintended pregnancy was found to be high during the study period. Factors associated with unintended pregnancy were no exposure to community education, no bad obstetric history, not being a primary decision maker for family planning, not having developed pregnancy-related complications before index-pregnancy and lack of health-care provider support
Burt [36]	Indirect effects of COVID-19 on maternal, neonatal, child, sexual and reproductive health services in Kampala, Uganda	BMJ global health	Cross sectional study	Uganda	Maternal, neonatal, child, sexual and reproductive health services	There were disruptions in antenatal and vaccination service during the lockdown. Response to COVID-19 resulted in an increase in pregnancy complications and fetal and infant outcomes. There were increases in high blood pressure cases among women, stillbirths, low-birthweight and premature infant births, the rate of neonatal unit admissions, neonatal deaths and abortions
Kassie et al. [37]	Impact of coronavirus diseases-2019 (Covid-19) on utilization and outcome of reproductive, maternal, and newborn health services at governmental health facilities in south west Ethiopia, 2020: Comparative cross-sectional study	International journal of women's health	Cross-sectional study	Ethiopia	Utilization of reproductive, maternal, and newborn health services	The pandemic led to reductions in utilization of reproductive, maternal and newborn health-services
Shakespeare et al. [38]	Resilience and vulnerability of maternity services in Zimbabwe: a comparative analysis of the effect of Covid-19 and lockdown control measures on maternal and perinatal outcomes, a single-centre cross-sectional study at Mpilo Central Hospital	BMC pregnancy & childbirth	Cross-sectional study	Zimbabwe	Maternal and perinatal morbidity and mortality	The lockdown did not result in any significant maternal and perinatal adverse outcomes
Shuka et al. [39]	Use of healthcare services during the COVID-19 pandemic in urban Ethiopia: evidence from retrospective health facility survey data	BMJ open	Retrospective study	Ethiopia	Maternal and child health services (family planning, antenatal and postnatal care, abortion care, delivery, and immunisation)	Utilization of several maternal and child health services remained unaffected, highlighting the resilience of the healthcare system during the pandemic
Wood et al. [40]	Need for and use of contraception by women before and during COVID-19 in four sub-Saharan African geographies: results from population-based national or regional cohort surveys	The lancet global health	Cross-sectional study	Multiple countries (Burkina Faso, Kenya, Congo DR, and Nigeria)	Contraceptive use	The early stages of the COVID-19 pandemic did not have the expected adverse impact on access to and use of contraceptive services by women
Adelekan et al. [41]	Effect of COVID-19 pandemic on provision of sexual and reproductive health services in primary health facilities in Nigeria: a cross-sectional study	Reproductive health	Cross sectional study	Nigeria	Provision of sexual and reproductive health services	There was a significant reduction in utilization of sexual and reproductive health services during the lockdown even though many of these facilities were opened
Atim et al. [42]	COVID-19 and Health Sector Development Plans in Africa: The Impact on Maternal and Child Health Outcomes in Uganda	Risk management and healthcare policy	Cross sectional study	Uganda	Maternal and Child Health Outcomes	The pandemic has negatively impacted immunization, antenatal, sexual, and reproductive health, emergency and obstetric, and postnatal care services. Specifically, there were declines in under-five vitamin A coverage, measles vaccination coverage, isoniazid preventive therapy coverage, and facility-based deliveries. Maternal and under-five deaths increased, and outreaches were rarely conducted in the lockdown period
Chelo et al. [43]	Impact and projections of the COVID-19 epidemic on attendance and routine vaccinations at a pediatric referral hospital in Cameroon	Archives de pédiatrie	Cross-sectional study	Cameroun	Attendance and routine vaccinations	The COVID-19 pandemic resulted in a significant decrease in in consultation and vaccination activities
De Waard et al. [44]	Maternal and neonatal outcomes of COVID-19 in a high-risk pregnant cohort with and without HIV	South African medical journal	Prospective cohort	South Africa	Maternal and neonatal outcomes of COVID-19	Among high-risk pregnant women, those with COVID-19 had a significant increased risk of maternal mortality compared with other deliveries. There was no significant difference in maternal/neonatal outcomes for people living HIV compared with those without HIV
Gebreegziabher et al. [45]	Assessment of maternal and child health care services performance in the context of COVID-19 pandemic in Addis Ababa, Ethiopia: evidence from routine service data	Reproductive Health	Cross-sectional study	Ethiopia	Maternal and child health care services (postnatal care visit, safe abortion care, vaccination, contraceptive acceptance)	The period during the pandemic saw declines in maternal and child health care services (new family planning visits, antenatal care, safe abortion care, vaccination)
Leight et al. [46]	Short-term effects of the COVID-19 state of emergency on contraceptive access and utilization in Mozambique	PLoS ONE	Cross-sectional study	Mozambique	Utilization of contraceptive health services	The period following the imposition of COVID-19 related measures saw a temporary modest decline in reproductive health service provision and utilization
Shapira et al. [47]	Disruptions in maternal and child health service utilization during COVID-19: analysis from eight sub-Saharan African countries	Health policy & planning	Retrospective study	Multiple countries (Cameroon, Congo DR, Liberia, Malawi, Mali Nigeria, Sierra Leone, and Somalia)	Maternal and child health service utilization	Service disruptions were experienced by all the countries. The most affected services were outpatient consultations and child vaccinations
Tadesse [48]	Antenatal Care Service Utilization of Pregnant Women Attending Antenatal Care in Public Hospitals During the COVID-19 Pandemic Period	International journal of women's health	Cross-sectional study	Ethiopia	Antenatal care utilization	Antenatal care utilization was negatively impacted by maternal age; residency status; educational status; still birth history; maternity service diversion and interruption, COVID-19 pandemic fear, and transportation challenges
Temesgen et al. [49]	Maternal health care services utilization amidst COVID-19 pandemic in West Shoa zone, central Ethiopia	PLoS ONE	Cross-sectional study	Ethiopia	Maternal health care services utilization	Maternal health care services utilization was low (64.8%) during the COVID-19 pandemic
Wanyana et al. [50]	Rapid assessment on the utilization of maternal and child health services during COVID-19 in Rwanda	Public Health Action	Cross-sectional study	Rwanda	Utilization of maternal and child health	There were declines in the utilization of maternal and child health services during the COVID-19 pandemic. The most affected areas were the Northern and Western provinces
Banke-Thomas et al. [51]	Utilization cost of maternity services for childbirth among pregnant women with coronavirus disease 2019 in Nigeria's epicenter	International journal of obstetrics and gynecology	Cross-sectional study (Hospital-based cost analysis)	Nigeria	Maternal services utilization costs	COVID-19 has directly impacted cost of maternity service utilization especially as governmental exemptions become unavailable, reduction of donations and presence of fees attributable to universal testing. Such huge costs of care may become unaffordable especially for pregnant women with COVID-19
Kayiga et al. [52]	Lived experiences of frontline healthcare providers offering maternal and newborn services amidst the novel corona virus disease 19 pandemic in Uganda: A qualitative study	PLOS ONE	Qualitative study (in-depth interviews)	Uganda	Lived experiences of frontline healthcare providers	There was a decline in the quality of maternal and newborn services during the pandemic. Barriers to service delivery included lack of transportation, fear of contracting COVID-19, salary cuts, loss of job etc. Facilitators of service delivery included passion to serve, availability of accommodation during the pandemic and others
Semaan et al. [53]	We are not going to shut down, because we cannot postpone pregnancy': a mixed-methods study of the provision of maternal healthcare in six referral maternity wards in four sub-Saharan African countries during the COVID-19 pandemic	BMJ global health	Mixed methods	Multiple countries (Guinea, Nigeria, Tanzania, and Uganda)	Provision of maternal healthcare	There was no change in the proportion of caesarean sections during the pandemic. Overall, provision of routine maternal care (childbirth care) was maintained in the six referral hospitals. However, challenges were reported for care provision to women suspected or confirmed with COVID-19

### Characteristics of included articles/studies

Three major themes were identified from the scoping review: 1) delayed or decreased care, 2) disruption in service provision and utilization and 3) mitigation strategies/recommendations.

Majority of the included studies (n = 18, 67%), used cross-sectional design. Among the included studies, most were conducted in Ethiopia (n = 7, 26%) as shown in Table 1.

#### Delayed or decreased care

Most studies reported delayed or decreased maternal and child health care services (n = 21, 78%) as a major impact of the COVID-19 pandemic. Delayed care-seeking behaviours for reproductive, maternal, newborn and child health services increased, along with an increased preference for home births. This behaviour change was reportedly due to fear of being exposed to COVID-19, reduced health system capacity, implemented COVID-19 mitigation strategies and restrictions, transport challenges and economic challenges from the pandemic [27–29]. This was especially true for refugee women due to the lack of inclusive public health policies, causing anxiety and low uptake of care [29].

Two sources reported a substantial decrease in-hospital care and admissions as a result of the COVID-19 mitigation strategies [27, 28]. Some women could not leave their homes due to the imposed lockdowns or lack of transportation to health care facilities [30]. One study reported a positive correlation between not attending health clinics and breast feeding [31]. Pandemic-related anxiety was also highlighted as a reason for delayed care or reduced service utilization by pregnant women and children [32]. Pregnant women in Nigeria commonly experienced stress and anxiety during the lockdown, underscoring the need for mental health screening during antenatal care to protect maternal health and ensure health care service utilization [33].

Delayed and decreased care can lead to more significant long-term disabilities and higher mortality rates in vulnerable populations. The decrease in hospital admission and increase in neonatal mortality rates were apparent during the pandemic [27], likely due to the disruptions of and decreased access to maternal and newborn care [34]. A 47 percent increase in facility neonatal deaths was reported in the first few months of the pandemic as a result of the disruption and diversion of health resources [28]. Delayed care may have also caused a spike in pregnancy complications, unintended pregnancies, an alarming increase in teenage abortions, cesarean section rates, and poor perinatal outcomes, calling for further investigation [35–37].

Interestingly, three studies indicated that utilization of services was unaffected by the pandemic due to the resiliency of the health care system [38–40].

#### Disruption in service provision and utilization

Disruption in service provision and utilization was a major theme illuminated in many of the included studies [28, 29, 32, 36, 37, 39, 41–50]. Clinic attendance and hospital admissions were significantly reduced for children under the age of five [28]. Some commonly affected health care services were childhood vaccinations, outpatient consultations and family planning services [43]. These disruptions further resulted in adverse health outcomes such as high blood pressure among women, stillbirths, low-birth weights, neonatal deaths and abortions [36]. Shapira and colleagues quantified maternal and child health disruptions in eight Sub-Saharan countries and concluded that child vaccinations and outpatient consults were most severely disrupted. In some countries, antenatal care, postnatal care and facility births also declined [47]. Cost was reported as another disruption to maternity services for childbirth. The cost for spontaneous vaginal birth and cesarean birth in Nigeria doubled or tripled relative to pre-pandemic expenditures [51].

Barriers to proper service delivery such as shorter patient consultation, failure to keep patient appointments, fear of contracting COVID-19 and limited access to personal protective equipment resulted in reduced quality of maternal and newborn care during the pandemic [48, 52]. Uganda was committed to serving their patients by implementing mitigation strategies, such as transportation and accommodation at health facilities, to support staff despite these reported barriers [52].

#### Mitigation strategies and recommendations

Studies offered strategies and measures to minimize the impact of the COVID-19 pandemic on maternal and child health and to address barriers that were reducing the utilization of maternal and child health services. It was suggested that communities should be educated about COVID-19 prevention and treatment, and maternity health services [35, 45, 53]. Promotion and education on these subjects, including antenatal care, the safety of in-facility births, and COVID-19 vaccinations are needed to ensure the continued use of health care services during the pandemic [45, 50]. The need to provide adequate resources for health care personnel, such as personal protective equipment, and the regulation of these resources was also highlighted [27, 51].

Authors illuminated the undeniable need to ensure women’s access to maternal and child health services during pandemics and lockdowns to protect the health of their babies and themselves [42, 48, 50]. In areas where early marriage, unexpected pregnancies and unsafe abortions are prevalent, it becomes crucial to continually provide MNCH services [42, 48] and provide proper transportation to these services during crises [48]. Both government and non-governmental agencies are needed to strengthen the delivery of and access to these services, and regulate the costs of health care procedures and personal protective equipment during current and future pandemics [41, 51].

There is a need to investigate the short-term and long-term impacts of the pandemic on maternal and child health outcomes [28, 42, 50] and for research to identify approaches to reduce the ongoing disruptions on maternal and child health in this region [39].

Lastly, it was suggested that African should continually build a resilient and sustainable health system as a strategy for providing uninterrupted MNCH services during future emerging diseases [39, 42].

## Discussion

As the COVID-19 pandemic continues to stretch the health care systems of many countries, we note a need to be mindful of its direct and indirect effects on maternal and child health care in Africa, given the already fragile health care systems across the continent. Africa constitutes about 11% of the world’s population but has 60% and 90% of the world’s population living with HIV/AIDS and Malaria respectively. The continent has the most maternal and child deaths due to severe and preventable illnesses each year, despite the significant gains for maternal and child outcomes in Africa over the past decade [54]. Nonetheless, the overall ramification of the pandemic in this region therefore, may be more protracted than other regions. These poor maternal and child health outcomes reflect the impact of poor health care financing, governance, and information systems, as well as poor access to medical supplies, vaccines, technology and service delivery.

Available publications included in this review indicate that time-sensitive programs such as maternity services, immunization and reproductive health services that are essential to the wellbeing of mothers and children have not been prioritized during the pandemic, although the findings of three studies showed otherwise in the case of Zimbabwe and elsewhere [38–40].

Although restrictions and other mitigation measures were expected to reduce the spread of the coronavirus, included studies noted poor access to care and low utilization of maternal and child health services, including reproductive services. Restricted availability and low uptake led to reduced reproductive health services, adverse health outcomes, declines in child vaccination, reduced antenatal care attendance and fewer hospital births, with simultaneous increases in home births and neonatal deaths [36, 43, 47].

The disproportionate impact of the pandemic on the health of the most vulnerable population segments is consistent with global reports which suggest that pregnant women, infants, children, and the elderly are more likely to be affected by infectious diseases than other people, especially in low-income countries. Moreover, a WHO report on acute respiratory infections suggests that emerging infections require prompt changes to treatment center plans to include, triage, repurposing, capacity building and awareness creation for personnel and clients to reduce public health risks and health disparity associated with the disease [55].

In Kenya, there was a report of increased preference for home-based births [29], which calls into question what strategies were most suited to safeguard the continued delivery of routine health programs while preventing the adverse effect of the pandemic on maternal and child health. Effective healthcare policies should be based on evidence-based guidelines and should focus on mitigating ongoing maternal and child health challenges, and providing continuous access to MNCH services, especially during global crises [42, 48, 50]. To improve the disproportionate access to health services, strategies should include education and training of the workforce and communities [45, 53], especially those working with the low- income areas, to enhance knowledge about emergency preparation, maternity and child health services, and strategies to alleviate the challenges posed by pandemics.

Clinical and public health research plays an important role in policy implementation during times such as the COVID-19 pandemic, and we believe that the WHO, Centers for Diseases Control and Prevention (CDC), Civil societies and other stakeholders have a role to play in monitoring the implementation of COVID-19 guidelines across jurisdictions. There is a need to sensitize corporate / international donors about the dangers of reducing their contributions during emergencies so that resources remain accessible during crises. It is important to involve key stakeholders at local and national levels, including government and non-governmental agencies to advocate for the proper allocation and regulation of available health resources [41].

The findings from this review highlight the importance of identifying strategies and guidelines that will allow MNCH services to remain accessible during emerging infectious diseases to protect the health of women and their babies. Further research on the short-term and long-term effects of the COVID-19 pandemic, and approaches to address barriers to MNCH services during the COVID-19 pandemic should address this gap [28, 42, 50, 56].

There are, however, gaps in the operationalization of MNCH programs and services across the region as shown in the studies reviewed. Lack of emergency preparedness plans for human and material resources tailored to peculiar health system gaps, and health needs remain an ongoing challenge in African regions. This weakness will need to be urgently addressed to reduce additional MNCH risks during global health emergencies.

The comprehensive and rigorous nature of our search strategy and scoping review methodology are major strengths of this review. The duplicate screening by two authors with MNCH expertise and review experience is another strength. With respect to study limitations, potential delay in indexing might have excluded some relevant studies. Also, we did not appraise the quality of the methodology of each study since our priority was to rapidly compile evidence of this evolving topic and our strategy was limited to English papers published between January 2020 and March 2022.

## Conclusions

This scoping review sought to document the effects that the COVID-19 pandemic has had on maternal and child health in relation to health care access, utilization, and health outcome in Africa. This review identified limited research on this topic, however, the findings suggest that despite the various mitigation strategies and recommendations adopted across jurisdictions, the impact on maternal and child health outcomes and health systems is cause for concern. The reduced access and utilization of maternal and prenatal care, midwifery practice and neonatal care, including poor access to sexual and reproductive health care, directly and indirectly impact maternal and child health outcomes. The implications include continuous disruptions in services and delayed or lack of access to needed care for women and children. Our review has shown that maternity services, immunization, and reproductive health services that promote the wellbeing of mothers and children have not been prioritized during the pandemic and need to be promoted during crises. There is a continuing need for health systems capacity expansion with built-in redundancies to shield the continent’s most vulnerable population segments from the shocks of the current COVID-19 pandemic and future global health emergencies.

## Supplementary Information

**Additional file 1**. Search strategy.

## Data Availability

Not applicable.

## Abbreviations

COVID-19: Coronavirus disease 2019
MNCH: Maternal newborn and child health
SARS-CoV-2: Severe acute respiratory syndrome coronavirus 2
WHO: World health organization
