# Supplementary material for: The direct and indirect impact of COVID-19 pandemic on maternal and child health services in Africa: a scoping review
Source: Glob Health Res Policy. 2022 Jul 20;7:20. doi: 10.1186/s41256-022-00257-z (PMC9296365; doi:10.1186/s41256-022-00257-z)
Supplement: Supplementary file 1 — Additional file 1. Search strategy. [file 41256_2022_257_MOESM1_ESM.docx]

**The direct and indirect impact of COVID-19 pandemic on maternal and child health services in Africa: a scoping review**

Prince A. Adu^1,2^, Lisa Stallwood^1^, Stephen O. Adebola^3,4^, Theresa Abah^5^, Arnold Ikedichi Okpani^1,6^

**Search Strategy**

**Database: Ovid MEDLINE**

**Date: March 15, 2022, 6:40 PM EST**

| 1. (Maternal and child health).mp. [mp=title, abstract, original title, name of substance word, subject heading word, floating sub-heading word, keyword heading word, organism supplementary concept word, protocol supplementary concept word, rare disease supplementary concept word, unique identifier, synonyms] | 17076 |
| --- | --- |
| 1. MNCH.mp. | 280 |
| 1. Maternal health.mp. or exp Maternal Health/ or exp Maternal Health Services/ | 62586 |
| 1. reproductive health.mp. or exp Reproductive Health/ or exp Reproductive Medicine/ | 42148 |
| 1. perinatal care.mp. or exp Perinatal Care/ | 13277 |
| 1. antenatal health.mp. or exp Prenatal Care/ | 30992 |
| 1. prenatal care.mp. or exp Prenatal Care/ | 37084 |
| 1. postnatal care.mp. or exp Postnatal Care/ | 7872 |
| 1. infant health.mp. or exp Infant Health/ | 4331 |
| 1. 1 or 2 or 3 or 4 or 5 or 6 or 7 or 8 or 9 | 122284 |
| 1. Covid-19.mp. or exp COVID-19/ | 226386 |
| 1. Covid19.mp. | 2355 |
| 1. exp Coronavirus Infections/ or covid-2019.mp. | 157014 |
| 1. sars-cov-2.mp. or exp SARS-CoV-2/ | 152039 |
| 1. severe acute respiratory syndrome.mp. or exp Severe Acute Respiratory Syndrome/ | 33673 |
| 1. coronavirus.mp. or exp Coronavirus/ | 179175 |
| 1. Severe acute respiratory syndrome coronavirus-2.mp. | 22924 |
| 1. 11 or 12 or 13 or 14 or 15 or 16 or 17 | 255362 |
| 1. Africa.mp. or exp Africa/ | 351729 |
| 1. (((Africa* or Algeria or Angola or Benin or Botswana or Burkina Faso or Burundi or Cabo Verde or Cameroon or Central African Republic or Chad or Comoros or Congo or Cote d'Ivoire or Djibouti or Egypt or Equatorial Guinea or Eritrea or Eswatinor Ethiopia or Gabon or Gambia or Ghana or Guinea or Guinea-Bissau or Kenya or Lesotho or Liberia or Libya or Madagascar or Malawi or Mali or Mauritania or Mauritius or Morocco or Mozambique or Namibia or Niger or Nigeria or Rwanda or Sao Tome) and Principe) or Senegal or Seychelles or Sierra Leone or Somalia or South Africa or South Sudan or Sudan or Tanzania or Togo or Tunisia or Uganda or Zambia or Zimbabwe).mp. [mp=title, abstract, original title, name of substance word, subject heading word, floating sub-heading word, keyword heading word, organism supplementary concept word, protocol supplementary concept word, rare disease supplementary concept word, unique identifier, synonyms] | 143623 |
| 1. 19 or 20 | 366888 |
| 1. 10 and 18 and 21 | 114 |
| 1. limit 22 to (English language and year="2020 -Current") | 113 |

**Database: Embase Classic+Embase**

**Date: March 15, 2022, 7:08 PM EST**

| 1. (Maternal and child health).mp. [mp=title, abstract, heading word, drug trade name, original title, device manufacturer, drug manufacturer, device trade name, keyword heading word, floating subheading word, candidate term word] | 19993 |
| --- | --- |
| 1. MNCH.mp. | 336 |
| 1. Maternal health.mp. or exp maternal welfare/ | 23661 |
| 1. Reproductive health.mp. or exp reproductive health/ | 30877 |
| 1. perinatal care.mp. or exp perinatal care/ | 67321 |
| 1. antenatal health.mp. or exp prenatal care/ | 170502 |
| 1. prenatal care.mp. or exp prenatal care/ | 173866 |
| 1. postnatal care.mp. or exp postnatal care/ | 133773 |
| 1. newborn care.mp. or exp newborn care/ | 46561 |
| 1. 1 or 2 or 3 or 4 or 5 or 6 or 7 or 8 or 9 | 389134 |
| 1. Covid-19.mp. or exp coronavirus disease 2019/ | 247228 |
| 1. Covid19.mp. | 4186 |
| 1. covid-2019.mp. or exp coronavirus disease 2019/ | 197442 |
| 1. sars-cov-2.mp. or exp Severe acute respiratory syndrome coronavirus 2/ | 102605 |
| 1. coronavirus.mp. or exp Coronavirinae/ | 252295 |
| 1. Severe acute respiratory syndrome coronavirus-2.mp. or exp Severe acute respiratory syndrome coronavirus 2/ | 68848 |
| 1. 11 or 12 or 13 or 14 or 15 or 16 | 282279 |
| 1. exp "Africa south of the Sahara"/ or exp South Africa/ or exp Africa/ or exp North Africa/ or exp Central Africa/ or Africa.mp. | 439551 |
| 1. (((Africa* or Algeria or Angola or Benin or Botswana or Burkina Faso or Burundi or Cabo Verde or Cameroon or Central African Republic or Chad or Comoros or Congo or Cote d'Ivoire or Djibouti or Egypt or Equatorial Guinea or Eritrea or Eswatinor Ethiopia or Gabon or Gambia or Ghana or Guinea or Guinea-Bissau or Kenya or Lesotho or Liberia or Libya or Madagascar or Malawi or Mali or Mauritania or Mauritius or Morocco or Mozambique or Namibia or Niger or Nigeria or Rwanda or Sao Tome) and Principe) or Senegal or Seychelles or Sierra Leone or Somalia or South Africa or South Sudan or Sudan or Tanzania or Togo or Tunisia or Uganda or Zambia or Zimbabwe).mp. [mp=title, abstract, heading word, drug trade name, original title, device manufacturer, drug manufacturer, device trade name, keyword heading word, floating subheading word, candidate term word] | 170004 |
| 1. 18 or 19 | 453607 |
| 1. 10 and 17 and 20 | 194 |
| 1. limit 21 to (English language and year="2020 -Current") | 194 |

**Database: Ovid HealthSTAR**

**Date: March 15, 2022, 7:38 PM**

| 1. (Maternal and child health).mp. [mp=title, abstract, original title, name of substance word, subject heading word, floating sub-heading word, keyword heading word, organism supplementary concept word, protocol supplementary concept word, rare disease supplementary concept word, unique identifier] | 16140 |
| --- | --- |
| 1. MNCH.mp. | 271 |
| 1. Maternal health.mp. or exp maternal welfare/ | 28097 |
| 1. Reproductive health.mp. or exp reproductive health/ | 18055 |
| 1. perinatal care.mp. or exp perinatal care/ | 12725 |
| 1. antenatal health.mp. or exp prenatal care/ | 29371 |
| 1. prenatal care.mp. or exp prenatal care/ | 34221 |
| 1. postnatal care.mp. or exp postnatal care/ | 7334 |
| 1. newborn care.mp. or exp newborn care/ | 1696 |
| 1. 1 or 2 or 3 or 4 or 5 or 6 or 7 or 8 or 9 | 93097 |
| 1. Covid-19.mp. or exp coronavirus disease 2019/ | 113446 |
| 1. Covid19.mp. | 963 |
| 1. covid-2019.mp. or exp coronavirus disease 2019/ | 111129 |
| 1. sars-cov-2.mp. or exp Severe acute respiratory syndrome coronavirus 2/ | 91314 |
| 1. coronavirus.mp. or exp Coronavirinae/ | 103401 |
| 1. Severe acute respiratory syndrome coronavirus-2.mp. or exp Severe acute respiratory syndrome coronavirus 2/ | 86940 |
| 1. 11 or 12 or 13 or 14 or 15 or 16 | 118929 |
| 1. exp "Africa south of the Sahara"/ or exp South Africa/ or exp Africa/ or exp North Africa/ or exp Central Africa/ or Africa.mp. | 276564 |
| 1. (((Africa* or Algeria or Angola or Benin or Botswana or Burkina Faso or Burundi or Cabo Verde or Cameroon or Central African Republic or Chad or Comoros or Congo or Cote d'Ivoire or Djibouti or Egypt or Equatorial Guinea or Eritrea or Eswatinor Ethiopia or Gabon or Gambia or Ghana or Guinea or Guinea-Bissau or Kenya or Lesotho or Liberia or Libya or Madagascar or Malawi or Mali or Mauritania or Mauritius or Morocco or Mozambique or Namibia or Niger or Nigeria or Rwanda or Sao Tome) and Principe) or Senegal or Seychelles or Sierra Leone or Somalia or South Africa or South Sudan or Sudan or Tanzania or Togo or Tunisia or Uganda or Zambia or Zimbabwe).mp. [mp=title, abstract, original title, name of substance word, subject heading word, floating sub-heading word, keyword heading word, organism supplementary concept word, protocol supplementary concept word, rare disease supplementary concept word, unique identifier] | 106957 |
| 1. 18 or 19 | 280842 |
| 1. 10 and 17 and 20 | 82 |
| 1. limit 21 to (English language and year="2020 -Current") | 80 |

**Database: Web of science**

**Date: March 15, 8 PM**

| 1. (((((((((ALL=(Maternal and child health)) OR ALL=(much)) OR ALL=(Maternal health)) OR ALL=(Maternal Health Services)) OR ALL=(reproductive health)) OR ALL=(perinatal care)) OR ALL=(antenatal health)) OR ALL=(prenatal care)) OR ALL=(postnatal care)) OR ALL=(infant health) | [100,983](https://www-webofscience-com.myaccess.library.utoronto.ca/wos/woscc/summary/6e21ad14-eea3-42f6-a395-3c41975a0c2c-2a1fe3d5/relevance/1) |
| --- | --- |
| 1. ((((((((((ALL=(Covid19)) OR ALL=(Covid-19)) OR ALL=(Coronavirus Infections)) OR ALL=(covid-2019)) OR ALL=(sars-cov-2)) OR ALL=(SARS-CoV-2)) OR ALL=(severe acute respiratory syndrome)) OR ALL=(Severe Acute Respiratory Syndrome)) OR ALL=(coronavirus)) OR ALL=(Coronavirus)) OR ALL=(Severe acute respiratory syndrome coronavirus-2) | [10,347](https://www-webofscience-com.myaccess.library.utoronto.ca/wos/woscc/summary/b85ccb5b-1e26-484a-922c-991311a153c9-2a1fe2fe/relevance/1) |
| 1. ((((((((((ALL= (Africa)) OR ALL=(Angola) OR ALL=(Benin) OR ALL=(Botswana) OR ALL=(Burkina) ALL=(Faso) OR ALL=(Burundi) OR ALL=(Cabo Verde) OR ALL=(Cameroon) OR ALL=(Central African Republic) OR ALL=(Chad) OR ALL=(Comoros) OR ALL=(Congo) OR ALL=(Cote d'Ivoire) OR ALL=(Djibouti) OR ALL=(Equatorial Guinea) OR ALL=(Eritrea) OR ALL=(Eswatini) OR ALL=(Ethiopia) OR ALL=(Gabon) OR ALL=(Gambia) OR ALL=(Ghana) OR ALL=(Guinea) OR ALL=(Guinea-Bissau) OR ALL=(Kenya) OR ALL=(Lesotho) OR ALL=(Liberia) OR ALL=(Madagascar) OR ALL=(Malawi) OR ALL=(Mali) OR ALL=(Mauritania) OR ALL=(Mauritius) OR ALL=(Mozambique) OR ALL=(Namibia) OR ALL=(Niger) OR ALL=(Nigeria) OR ALL=(Rwanda) OR ALL=(Sao Tome) OR ALL=(Principe) OR ALL=(Senegal) OR ALL=(Seychelles) OR ALL=(Sierra Leone) OR ALL=(Somalia) OR ALL=(South Africa) OR ALL=(South Sudan) OR ALL=(Sudan) OR ALL=(Tanzania) OR ALL=(Togo) OR ALL=(Uganda) OR ALL=(Zambia) OR ALL=(Zimbabwe) | [345,402](https://www-webofscience-com.myaccess.library.utoronto.ca/wos/woscc/summary/af7b4095-866d-4662-9444-90a22c07d098-2a1fe229/relevance/1) |
| 1. #1 AND #2 AND #3 | [22](https://www-webofscience-com.myaccess.library.utoronto.ca/wos/woscc/summary/02a94554-74eb-4078-8384-589b41f30406-2a1fe570/relevance/1) |

**Database:** Scopus

**Date: March 15, 8:25 PM**

| 1. ( maternal AND child AND health ) OR ( maternal AND health ) OR ( maternal AND health AND services ) ( reproductive AND health ) OR ( perinatal AND care ) OR ( antenatal AND health ) OR ( prenatal AND care ) OR ( postnatal AND care ) OR ( infant AND health ) | 523,639 |
| --- | --- |
| 1. ( covid19 ) OR ( covid-19 ) OR ( coronavirus AND infections ) OR ( covid-2019 ) OR ( sars-cov-2 ) OR ( sars-cov-2 ) OR ( severe AND acute AND respiratory AND syndrome ) OR ( severe AND acute AND respiratory AND syndrome ) OR ( coronavirus ) OR ( coronavirus ) OR ( severe AND acute AND respiratory AND syndrome AND coronavirus-2 ) | 647,637   \|  \| \| --- \| |
| 1. africa* OR algeria OR angola OR benin OR botswana OR burkina AND faso OR burundi OR cabo AND verde OR cameroon OR central AND african AND republic OR chad OR comoros OR congo OR cote AND d'ivoire OR djibouti OR egypt OR equatorial AND guinea OR eritrea OR eswatini AND ethiopia OR gabon OR gambia OR ghana OR guinea OR guinea-bissau OR kenya OR lesotho OR liberia OR libya OR madagascar OR malawi OR mali OR mauritania OR mauritius OR morocco OR mozambique OR namibia OR niger OR nigeria OR rwanda OR "Sao Tome and Principe" OR senegal OR seychelles OR sierra AND leone OR somalia OR south AND africa OR south AND sudan OR sudan OR tanzania OR togo OR tunisia OR uganda OR zambia OR zimbabwe | 1,665   \|  \| \| --- \| |
| 1. #1 AND #2 AND #3 | 49 |
| 1. #4 ( LIMIT-TO ( PUBYEAR , 2021 ) OR LIMIT-TO ( PUBYEAR , 2020 ) OR (PUBYEAR, 2022) ) AND ( LIMIT-TO ( LANGUAGE , "English" ) ) | 8 |

**Database: PubMed**

**Date: March 15, 2022, 8:44 PM**

| 1. ( ( maternal AND child AND health ) OR ( maternal AND health ) OR ( maternal AND health AND services ) ( reproductive AND health ) OR ( perinatal AND care ) OR ( antenatal AND health ) OR ( prenatal AND care ) OR ( postnatal AND care ) OR ( infant AND health ) ) AND ( ( covid19 ) OR ( covid-19 ) OR ( coronavirus AND infections ) OR ( covid-2019 ) OR ( sars-cov-2 ) OR ( sars-cov-2 ) OR ( severe AND acute AND respiratory AND syndrome ) OR ( severe AND acute AND respiratory AND syndrome ) OR ( coronavirus ) OR ( coronavirus ) OR ( severe AND acute AND respiratory AND syndrome AND coronavirus-2 ) ) AND ( africa* OR algeria OR angola OR benin OR botswana OR burkina AND faso OR burundi OR cabo AND verde OR cameroon OR central AND african AND republic OR chad OR comoros OR congo OR cote AND d'ivoire OR djibouti OR egypt OR equatorial AND guinea OR eritrea OR eswatini AND ethiopia OR gabon OR gambia OR ghana OR guinea OR guinea-bissau OR kenya OR lesotho OR liberia OR libya OR madagascar OR malawi OR mali OR mauritania OR mauritius OR morocco OR mozambique OR namibia OR niger OR nigeria OR rwanda OR "Sao Tome and Principe" OR senegal OR seychelles OR sierra AND leone OR somalia OR south AND africa OR south AND sudan OR sudan OR tanzania OR togo OR tunisia OR uganda OR zambia OR zimbabwe )) | 69 |
| --- | --- |
| 1. **#1** ( LIMIT-TO ( PUBYEAR , 2021 ) OR LIMIT-TO ( PUBYEAR , 2020 ) OR (PUBYEAR, 2022) ) AND ( LIMIT-TO ( LANGUAGE , "English" ) ) | 61 |
